# Acute Portal Vein Thrombosis Treated with Recombinant Human Soluble Thrombomodulin Combined with Antithrombin III

**DOI:** 10.1155/2020/8268016

**Published:** 2020-04-13

**Authors:** Satoshi Nakayama, Naoya Murashima

**Affiliations:** Department of Gastroenterology, Mishuku Hospital, 5-33-12, Kamimeguro, Meguro-ku, Tokyo 153-0051, Japan

## Abstract

Portal vein thrombosis is a major complication associated with liver cirrhosis. In cirrhotic patients, a decrease in procoagulant and anticoagulant factors and an unstable balance between them is observed, and a relative decrease in the activation of anticoagulant drivers is one of the main causes of portal vein thrombosis (PVT). Herein, we report a case of acute portal thrombosis associated with liver cirrhosis and treated with a recombinant form of soluble thrombomodulin (thrombomodulin alpha, TM-*α*) in combination with antithrombin III. TM-*α* was administered in accordance with the dosage and route of administration for disseminated intravascular coagulation therapy and resulted in dissolution of PVT with a gradual decrease in D-dimer levels. No adverse events were observed during the course of treatment. In the future, in addition to conventional anticoagulation therapy using heparin or antivitamin K drugs, novel therapies targeting protein C activation using a recombinant form of soluble thrombomodulin may play an important role in the treatment of acute PVT.

## 1. Introduction

Portal vein thrombosis (PVT) is frequently associated with liver cirrhosis, with a prevalence rate of approximately 1% among compensated cirrhotic patients and 8% to 25% among candidates for liver transplantation [1, 2]. Gastrointestinal bleeding, development or abrupt worsening of ascites, or hepatic encephalopathy are occasionally associated with the onset of PVT [3]. Decreased portal blood flow and reduced serum levels of endogenous coagulation inhibitors, such as protein C, protein S, and antithrombin III (AT III), are presumed to be the main factors involved in PVT [4, 5], and low-molecular weight heparin, heparinoid, and vitamin K antagonists are conventionally used as anticoagulant treatments [5, 6]. Thrombomodulin is a vascular endothelial cell surface protein that forms a complex with thrombin and inhibits its activity in addition to activating protein C [7, 8]. Recently in Japan, a recombinant form of soluble thrombomodulin (thrombomodulin alpha, TM-*α*) has been used as an anticoagulant for disseminated intravascular coagulation (DIC) following the demonstration of its noninferiority and safety relative to heparin therapy [9, 10]. TM-*α* may also be beneficial as an anticoagulant for the treatment of PVT.

## 2. Case Presentation

**Figure 1:**
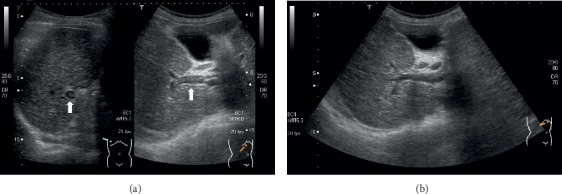
Ultrasonogram of the liver. (a) Before treatment: portal thrombus was seen in the right portal branch (arrow). (b) The thrombus was almost completely dissolved on day 6.

**Figure 2:**
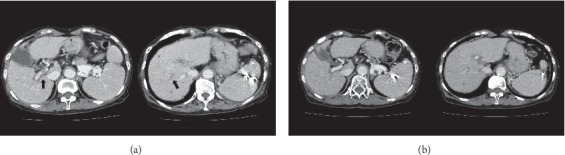
CT images of the liver. (a) Before treatment: portal thrombi were locally scattered in the right portal branch (arrow). (b) The thrombi were not seen on day 16.

**Figure 3:**
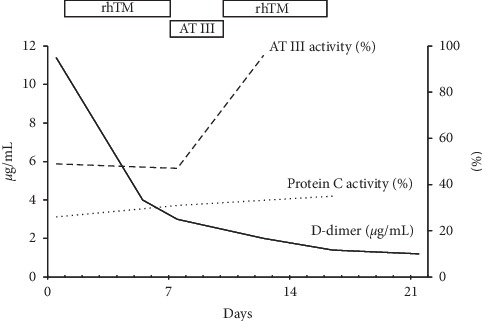
Changes in coagulation-related values. rhTM: recombinant human soluble thrombomodulin. AT III : antithrombin III.

A 79-year-old Japanese female, an HCV-related cirrhotic patient, was admitted to our hospital for general malaise with mild fever, and she was newly diagnosed with acute PVT based on ultrasonography results. The thrombi were locally scattered in the right branches, and hepatocellular carcinomas and ascites were not seen (Figures 1 and 2). The patient had a history of esophageal variceal bleeding. A blood test at the time of admission showed the following results (Table 1): hemoglobin, 10.5 g/dl; white blood cell count, 3590/*μ*L; platelet count, 10.0 × 10^4^/*μ*L; prothrombin time international normalized ratio, 1.22 (normal value 0.85–1.10); D-dimer, 11.4 *μ*g/mL (normal 0.0–1.0); activated protein C, 26% (normal 64–146); total protein S, 49% (normal 65–135); activated antithrombin III (AT III), 49% (normal 79–121); aspartate aminotransferase (AST), 47 IU/L; alanine aminotransferase (ALT), 29 IU/L; total bilirubin, 1.3 mg/dL; albumin, 2.3 g/dL; and creatinine, 1.02 mg/dL. The Child-Pugh score was 7 (Grade B), and the MELD score was 10. Because the patient had a history of variceal bleeding and DIC from an infection of unknown origin could not be completely ruled out, we chose a short course of anticoagulant therapy with thrombomodulin alpha (TM-*α*: Recomodulin^TM^, Asahi Kasei Pharma, Tokyo), a recombinant human soluble thrombomodulin, rather than with a heparin preparation or vitamin K antagonist. Informed consent was obtained following explanation of the administration procedure. We initially administered TM-*α* intravenously at a dose of 12800 U (standard dose 130–380 U/kg/day) in a daily single drip intravenous injection for 6 consecutive days in accordance with the dosage and route of administration for DIC. After the treatment was started, the serum level of D-dimer gradually declined (Figure 3) and the thrombus was almost completely dissolved (Figure 1). Due to the observation of a tendency for thrombolysis and a low serum level of AT III, we also injected 1500 IU of human AT III for 3 consecutive days, and sequentially, same dose of TM-*α* for further 6 days. The level of D-dimer subsequently declined further (Figure 3), and the thrombus completely disappeared (Figure 2). Portal vein thrombosis did not relapse thereafter, and known rare adverse events associated with TM-*α* therapy, such as intracranial, gastrointestinal, or pulmonary hemorrhage, were not observed during the treatment. After that, while no recurrence prevention treatment had been performed, a new PVT did not recur for one year or more.

## 3. Discussion

For this case of cirrhosis with acute PVT, in consideration of the possibility of a rapid reduction of portal venous flow and aggravation of collateral flow to esophageal varices, we performed anticoagulation therapy. While anticoagulation therapy for PVT in cirrhotic patients has been controversial [4], a recent systematic review and meta-analysis revealed a rate of portal vein recanalization ranging from 37% to 93%, a complete recanalization rate of 0% to 75%, and an anticoagulation-related bleeding rate of 0% to 18%. It was therefore concluded that anticoagulation therapy increases recanalization and reduces the progression of thrombosis compared with no therapy [11]. Anticoagulation drugs have been used based on empirical evidence for anticoagulation therapy of acute PVT in the clinical setting, including heparin, low-molecular weight heparin, and heparin-like analogues, which activate AT III, and warfarin, which inhibits vitamin K-dependent coagulation factor activity [12]. Recently, the effect of monotherapy with antithrombin-III for PVT has also been reported [13]. The effectiveness of new anticoagulation drugs, such as direct-acting antithrombin drugs or anticoagulation factor Xa drugs, is still unknown [12].

According to recent research, procoagulation and anticoagulation factors are reduced in parallel, resulting in a rebalancing of coagulation in cirrhotic patients. However, the coagulation balance is fragile and can be tipped toward hemorrhage or thrombosis, depending on the prevailing circumstantial risk factors [14]. Because of a decrease in anticoagulant factors such as antithrombin and protein C, and an increase in factor VIII, a tendency for dominance of a prohemostatic state is typically found in cirrhotic patients, particularly those with portal vein thrombus [14]. Thrombomodulin, a glycoprotein present on vascular endothelial cells, is involved in protein C activation in the natural coagulation cascade. Thrombomodulin reversibly binds to thrombin and downregulates thrombin, and the thrombomodulin-thrombin complex subsequently activates protein C. Activated protein C then suppresses the generation of thrombin by inactivating the activated coagulation factor V and activated factor VIII [15, 16]. Furthermore, the lectin-like domain of thrombomodulin has been shown to adsorb several inflammatory mediators. A recent report suggested that intrinsic resistance to the anticoagulant action of thrombomodulin is associated with de novo portal vein thrombosis and a low survival rate in patients with cirrhosis [17]. Thus, thrombomodulin and protein C-mediated activation of anticoagulant drivers may represent a new framework for anticoagulation therapy.

We administered TM-*α*, the world's first recombinant form of human thrombomodulin, which acts on coagulation and inflammation to regulate the anticoagulant cascade. Since TM-*α* therapy significantly improved DIC and alleviated bleeding symptoms as compared with heparin therapy in a Japanese phase III clinical trial of DIC patients, TM-*α* has been widely used to treat patients with DIC in Japan [9, 10, 18, 19]. In the present case, PVT disappeared following TM-*α* therapy in accordance with the dosage and route of administration for DIC, while there is no reported evidence about the treatment of portal vein thrombosis with TM-*α* this time. Although we also administered human AT III in the middle of the treatment course, a decrease of D-dimer and dissolution of the PVT were already seen after administration of TM-*α* alone. Thus, it appears that a sufficient thrombolytic effect was achieved with TM-*α* monotherapy. Although the therapeutic effect of TM-*α* administration may seem paradoxical because the production of protein C itself is reduced in patients with cirrhosis, in vitro data show that if protein C activity is 10% or greater, TM-*α* can inhibit the generation of thrombin [20].

Herein, we presented a case of liver cirrhosis in which PVT was safely treated with TM-*α*. Because this was only a single case experience, the response to treatment for PVT with TM-*α* relative to other conventional drugs remains unclear. In the future, however, a novel therapeutic approach targeting the activation of protein C with a recombinant form of soluble thrombomodulin may play an important role in the treatment of PVT.

## Figures and Tables

**Table 1:** A blood chemistry data at the time of admission.

RBC	357 × 10^4^/*μ*L
Hb	10.5 g/dL
Ht	32.3%
WBC	3590 *μ*L
Plt	10.0 × 10^4^/*μ*L
PT-INR	1.22
APTT	42.8 sec
Fibrinogen	172 mg/dL
FDP	21 *μ*g/mL
D-dimer	11.4 *μ*g/mL
Activated Protein C	26%
Total Protein S	46%
Activated AT-III	49%
AST	47 IU/L
ALT	29 IU/L
ALP	550 IU/L
*γ*-GTP	16 IU/L
T-Bil	1.3 mg/dL
Alb	2.3 g/dL
BUN	25.4 mg/dL
Cre	1.02 mg/dL
Ammonia	75 *μ*g/dL
Na	134 mEq/L
K	4.0 mEq/L
Cl	108 mEq/L
CRP	0.52 mg/dL
HBs-Ag	(−)
Anti-HCV	(+)
